# Function of FlhB, a Membrane Protein Implicated in the Bacterial Flagellar Type III Secretion System

**DOI:** 10.1371/journal.pone.0068384

**Published:** 2013-07-11

**Authors:** Vladimir A. Meshcheryakov, Clive S. Barker, Alla S. Kostyukova, Fadel A. Samatey

**Affiliations:** 1 Trans-Membrane Trafficking Unit, Okinawa Institute of Science and Technology, Okinawa, Japan; 2 School of Chemical Engineering and Bioengineering, Washington State University, Pullman, Washington, United States of America; Centro Nacional de Biotecnologia - CSIC, Spain

## Abstract

The membrane protein FlhB is a highly conserved component of the flagellar secretion system, and it plays an active role in the regulation of protein export. In this study conserved properties of FlhB that are important for its function were investigated. Replacing the *flhB* gene (or part of the gene) in *Salmonella typhimurium* with the *flhB* gene of the distantly related bacterium *Aquifex aeolicus* greatly reduces motility. However, motility can be restored to some extent by spontaneous mutations in the part of *flhB* gene coding for the cytoplasmic domain of *Aquifex* FlhB. Structural analysis suggests that these mutations destabilize the structure. The secondary structure and stability of the mutated cytoplasmic fragments of FlhB have been studied by circular dichroism spectroscopy. The results suggest that conformational flexibility could be important for FlhB function. An extragenic suppressor mutation in the *fliS* gene, which decreases the affinity of FliS to FliC, partially restores motility of the FlhB substitution mutants.

## Introduction

The bacterial flagellum is a large, complex molecular machine made up of more than 30 different proteins. It contains three major substructures: the basal body, the hook and the filament. Most of the flagellar proteins are localized outside of the cell and are exported across the cytoplasmic membrane by the flagellum-specific secretion apparatus. This apparatus is evolutionarily related to the type III secretion system that is used by many pathogenic bacteria for secretion of virulence factors into the host eukaryote cell cytoplasm [1], [2].

In the case of *Salmonella enterica* serovar Typhimurium (*S. typhimurium*), the flagellar secretion system consists of six integral membrane proteins: FlhA, FlhB, FliO, FliP, FliQ and FliR; and three cytoplasmic proteins: FliH, FliI, and FliJ [3]. Protein export by the flagellar type III secretion system is highly regulated. When the hook reaches an appropriate length (55.0±5.9 nm in *Salmonella* [4]), the secretion system switches substrate specificity from rod/hook-type export to filament-type export [3], [5]. Two proteins, the membrane protein FlhB and the hook-length control protein FliK, are critical for the substrate switching.

FlhB consists of two domains: a hydrophobic N-terminal part (FlhB_TM_) that is predicted to contain four transmembrane helices, and a C-terminal cytoplasmic domain (FlhB_C_) [6]. The two domains are connected by a flexible linker. The linker is important for FlhB function: its deletion or point mutations altering its flexibility have a significant effect on substrate secretion [7], [8]. The cytoplasmic domain of wild-type *Salmonella* FlhB undergoes autocatalytic cleavage between amino-acid residues Asn269 and Pro270 within a highly conserved Asn-Pro-Thr-His sequence [8], [9]. This auto-cleavage is essential for the switching process [7], [10]. Mutation of Asn269 to Ala prevents cleavage and locks the export apparatus in the rod/hook-type specificity state.

To switch substrate specificity, FlhB receives a signal from FliK [11], [12]. In the case of a deleted *fliK* gene the substrate switching does not occur and this results in a very long hook, termed “polyhook”, without any filament attached [4]. Several extragenic suppressor mutations, which allow the switching even in the absence of FliK, have been isolated and mapped to the part of *flhB* gene coding for FlhB_C_ [11], [13].

In the current work, to reveal functionally important properties of FlhB, we replaced the *flhB* gene of *Salmonella* with the *flhB* gene of *A. aeolicus,* or with a fusion gene encoding a chimera FlhB composed of FlhB_TM_ of *S. typhimurium* and FlhB_C_ of *A. aeolicus*. All substitution mutants were substantially less motile than wild-type cells. However, several spontaneous mutations were found altering the C-terminal part of FlhB and resulting in enhanced motility. The sites of these mutations were mapped onto the structure of *Aquifex* FlhB_C_ that was recently solved by X-ray crystallography [14], [15], [16]. Structural analysis suggested that the suppressor mutations destabilize the structure of FlhB_C_. The secondary structure and the stability of the mutated *Aquifex* FlhB_C_ protein were studied by circular dichroism spectroscopy. We conclude that conformational flexibility of the cytoplasmic part of FlhB could be important for its function. Additionally, an extragenic bypass mutation in the *fliS* gene that partially restores motility of FlhB substitution mutants has been found. This mutation affects affinity of FliS to FliC but not to FlhB.

## Results

### Design of *flhB* Fusion Genes Encoding FlhB Chimeras

**Figure 1:**
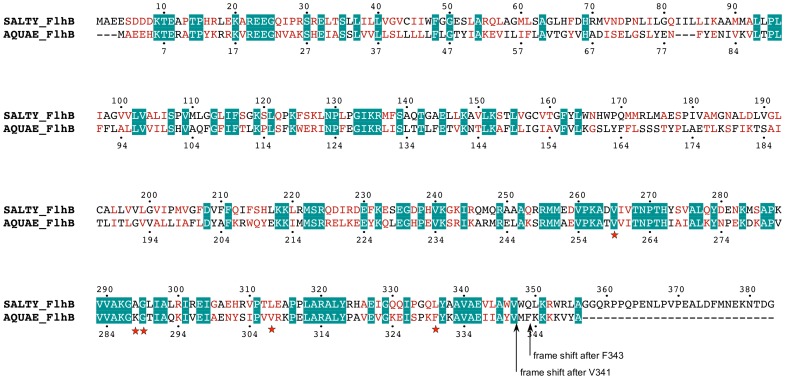
Amino acid sequence alignment of FlhB from *S. typhimurium* (SALTY_FlhB) and *A. aeolicus* (AQUAE_FlhB). Identical residues are shown with a teal background; similar residues are colored red. Suppressor mutations found in this study are marked by red stars (for point mutations) and black arrows (for frame shift mutations).

**Figure 2:**
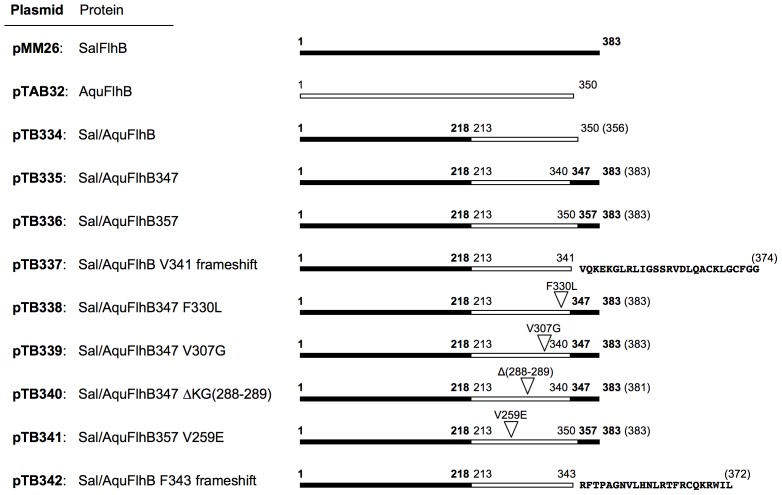
Schematic representations of FlhB products encoded by the plasmids used for the motility assay. Black and white bars indicate the *Salmonella* and *Aquifex* parts of FlhB, respectively. Triangles mark the positions of point and deletion suppressor mutations. Numbers in parentheses show the total number of amino acids in each protein.

*Aquifex* FlhB shares a 32% sequence identity with FlhB of *Salmonella* (Figure 1). To investigate the ability of the cytoplasmic domain from *Aquifex* FlhB to function within the *Salmonella* flagellar export apparatus, we constructed three genes to produce chimeric FlhB proteins where the N-terminal transmembrane region of *Salmonella* FlhB was fused to the C-terminal cytoplasmic domain of *Aquifex* FlhB. All of these chimeric FlhB proteins differed in their C-terminal sequences and have different levels of sequence homology to wild-type *Salmonella* FlhB (Figure 2).

The first chimera, Sal/AquFlhB, consisted of *Salmonella* FlhB_TM_ (residues 1–218) fused to *Aquifex* FlhB_C_ (residues 213–350). The C-terminus of *Aquifex* FlhB is shorter in comparison to *Salmonella* FlhB by 33 residues. Therefore, we also produced chimeric FlhB constructs with C-termini having the extra 33 residues just like in the case of wild-type *Salmonella* FlhB. One of these, Sal/AquFlhB357, consisted of *Salmonella* FlhB_TM_ fused to *Aquifex* FlhB_C_ followed by C-terminal residues 357–383 of *Salmonella* FlhB. Another chimera, Sal/AquFlhB347, consisted of *Salmonella* FlhB_TM_ fused to truncated *Aquifex* FlhB_C_ (residues 213–340) followed by C-terminal residues 347–383 from *Salmonella* FlhB.

**Figure 3:**
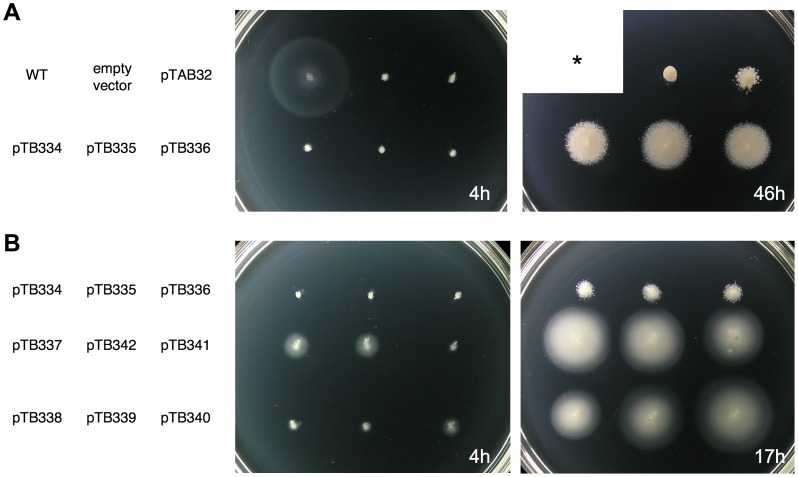
Swimming motility assay. (A) Motility of Δ*flhB Salmonella* strain transformed with plasmids coding for different variants of FlhB. The asterisk “*” marks the previous emplacement for the wild type strain that was removed because it would have overgrown after 46 hours. (B) Rescue of motility by mutations in the cytoplasmic domain of FlhB. The plates were incubated at 30°C for the indicated time. Indicated plasmids carry genes of the following proteins: wild-type SalFlhB (WT); AquFlhB (pTAB32); Sal/AquFlhB (pTB334); Sal/AquFlhB347 (pTB335); Sal/AquFlhB357 (pTB336); Sal/AquFlhB V341 frame-shift (pTB337); Sal/AquFlhB F343 frame-shift (pTB342); Sal/AquFlhB357 V259E (pTB341); Sal/AquFlhB347 F330L (pTB338); Sal/AquFlhB347 V307G (pTB339); Sal/AquFlhB347 ΔKG(288–289) (pTB340).

pTrc99A-FF4-based plasmids [17] expressing the different variants of FlhB proteins were transformed into Δ*flhB Salmonella* cells and tested for their ability to restore motility. Transformants were inoculated into tryptone soft agar plates. We found that all transformants were substantially less motile than wild-type cells (Figure 3A).

**Figure 4:**
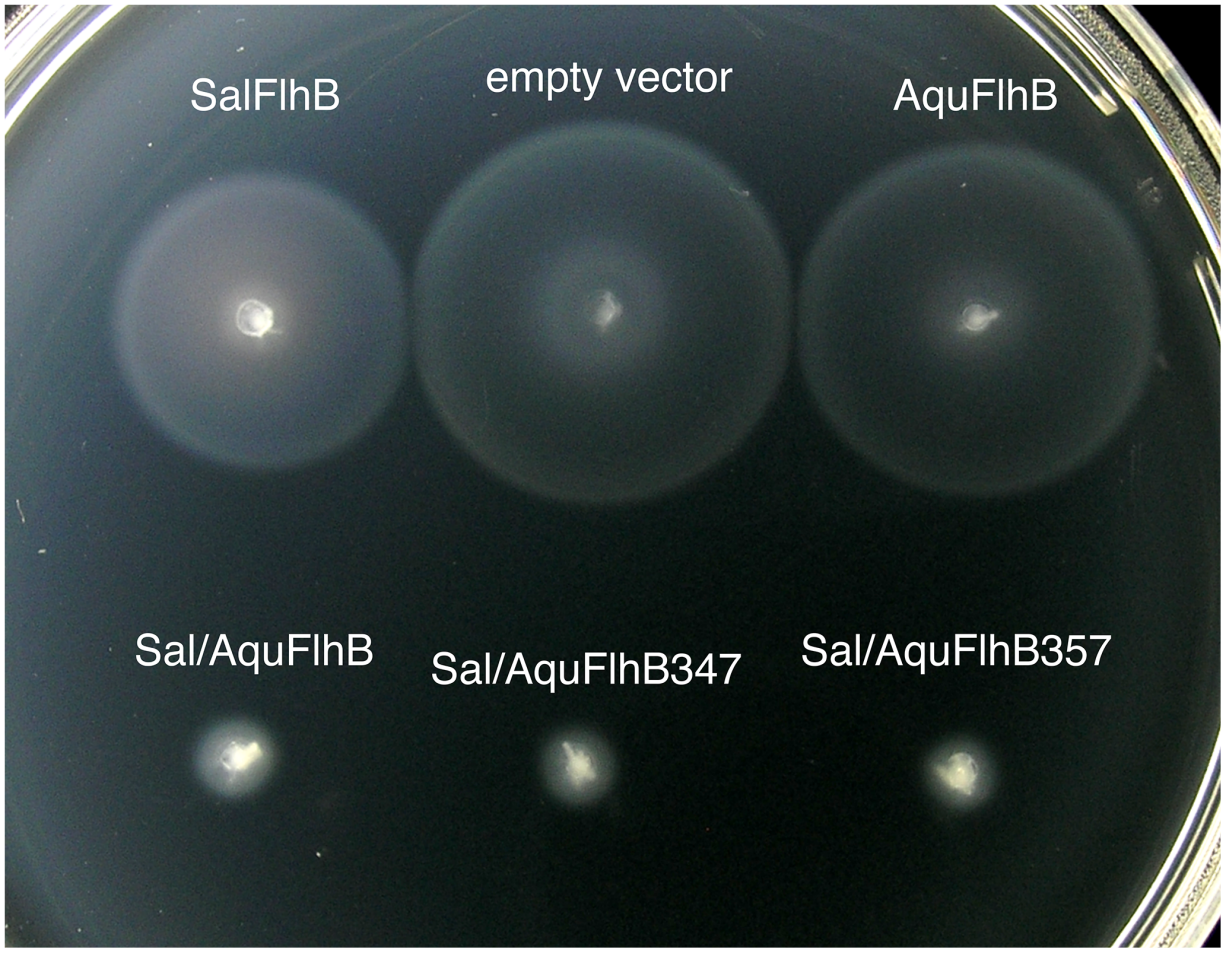
Dominance effect on motility of wild-type *Salmonella* strain SJW1103 transformed with the plasmids encoding different FlhB proteins. Soft agar plate containing 0.1 mM IPTG was incubated at 30°C for 5 hours.

To examine negative dominant effects on the motility of the wild-type cells, we tested the swimming of *Salmonella* wild-type strain SJW1103 transformed with the pTrc99A-based plasmids (Figure 4). All chimera proteins (Sal/AquFlhB, Sal/AquFlhB347, and Sal/AquFlhB357) inhibited motility of the wild-type cells. This finding suggests that these proteins could be incorporated into the *Salmonella* export apparatus. In contrast, motility of SJW1103 cells producing wild-type *Aquifex* FlhB was the same as that of the cells with the vector control, demonstrating that *Aquifex* FlhB cannot efficiently compete with the *Salmonella* protein. These results indicate the importance of the transmembrane region for FlhB to be inserted into secretion system.

### Isolation of Suppressor Mutants from Δ*flhB* Strains Producing Chimeric FlhB Proteins

After extended incubation, cells expressing chimeric *flhB* genes gave rise to suppressor mutants with enhanced motility, although motility was less than of the wild-type strain (Figure 3B).

To determine the positions of the suppressor mutations, the plasmids carrying the *flhB* genes that were initially transformed in Δ*flhB Salmonella* cells, were isolated and sequenced. Most of the suppressor mutations were located at the 3′-end of chimeric *flhB* gene, which encodes the C-terminal cytoplasmic part of FlhB. These mutations lead to single amino acid substitutions (V259E, V307G, and F330L), frame shifts (after V341 and F343), and an amino acid deletion (ΔKG 288–289) (Figure 2). Mutation ΔKG was encountered several times.

To confirm the effects of the found mutations on motility, isolated plasmids with the mutated *flhB* genes were re-transformed in Δ*flhB Salmonella* cells. All re-transformants, except one (see below), showed the same motility phenotype as originally obtained pseudorevertants (data not shown).

### Effect of the Suppressor Mutations on FlhB Autocleavage

**Figure 5:**
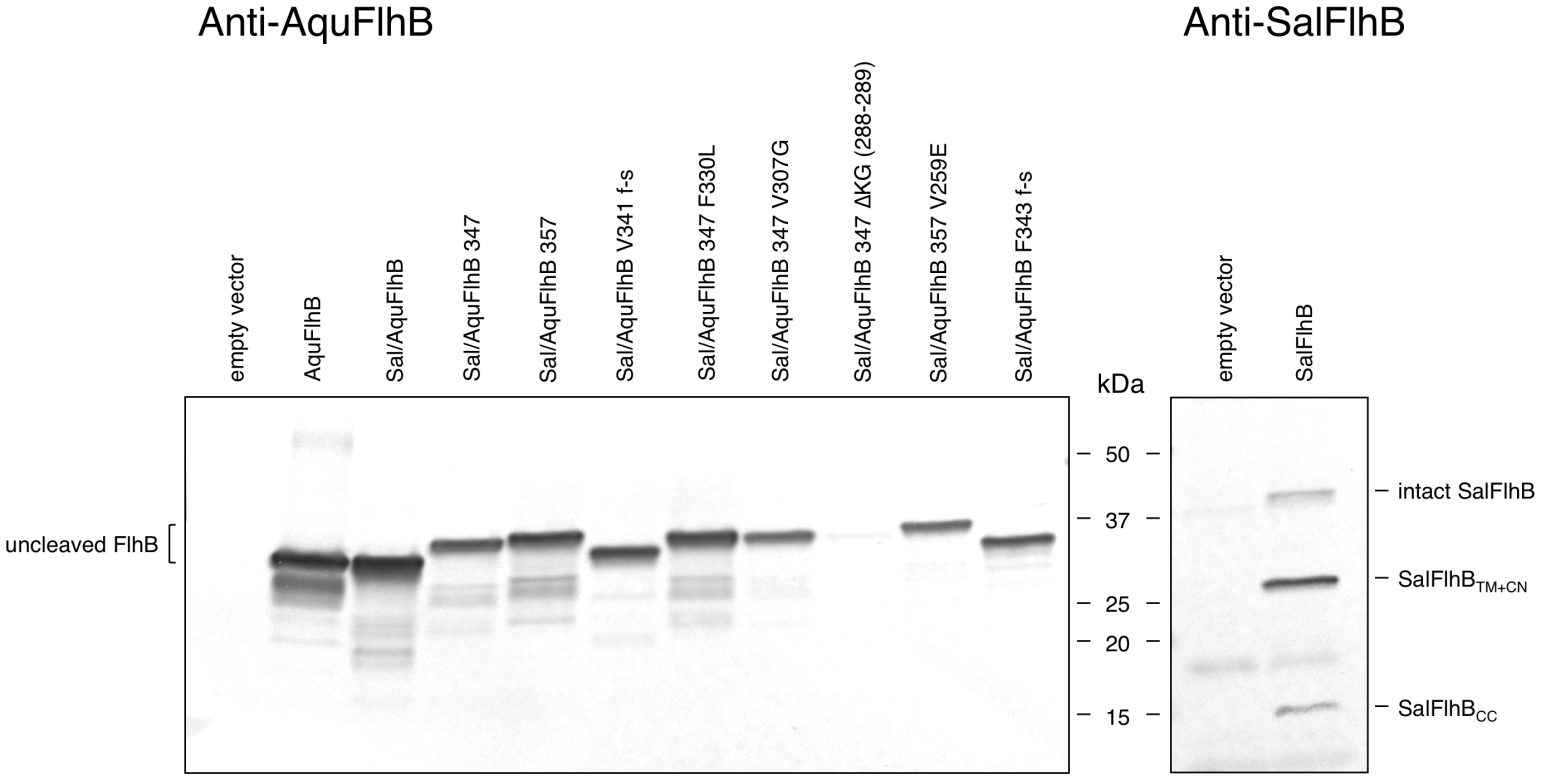
Autocleavage properties of FlhB. Cells carrying the plasmids encoding different FlhB proteins were grown at 37°C until OD_600_ = 0.5–0.6. Protein expression was induced by 0.1 mM IPTG. After 2 hours cells were harvested by centrifugation and analyzed by immunoblotting using polyclonal antibodies against full-length *Aquifex* and *Salmonella* FlhB.

The cytoplasmic domain of FlhB undergoes autocatalytic cleavage that is important for its function [10]. To examine whether the suppressor mutations affect FlhB cleavage, we performed affinity western blotting analysis with anti-SalFlhB and anti-AquFlhB polyclonal antibodies (Figure 5). In the case of wild-type *Salmonella* FlhB three bands were detected that correspond to uncleaved protein and the cleavage products. For *Aquifex* FlhB, the FlhB chimeras, and the suppressor mutants only uncleaved proteins could be identified (Figure 5). This does not mean that autocleavage of AquFlhB_C_ does not occur at all. All the *Aquifex* FlhB_C_ proteins we purified were fully cleaved (data not shown). Furthermore, the *Salmonella* cells carrying the chimera genes were motile and this also suggests cleavage occurred, since without autocleavage of FlhB switching of substrate specificity (and therefore motility) is not possible [7]. This indicates that the cytoplasmic domain of *Aquifex* FlhB was more stable within *Salmonella* cells, which might be because *Salmonella* is a mesophilic bacterium and has a lower growth temperature than the hyperthermophile *Aquifex*.

In the case of ΔKG (288–289) mutant, the protein was detected at very low level that could be explained by its instability or reduced affinity of polyclonal anti-*Aquifex* FlhB antibodies to this mutant.

### Mapping of the Suppressor Mutations into the Structure of FlhB_C_

The structures of *Salmonella* and *Aquifex* FlhB_C_ are available in the Protein Data Bank (PDB) with the accession numbers 3B0Z and 3B1S, respectively [16].

**Figure 6:**
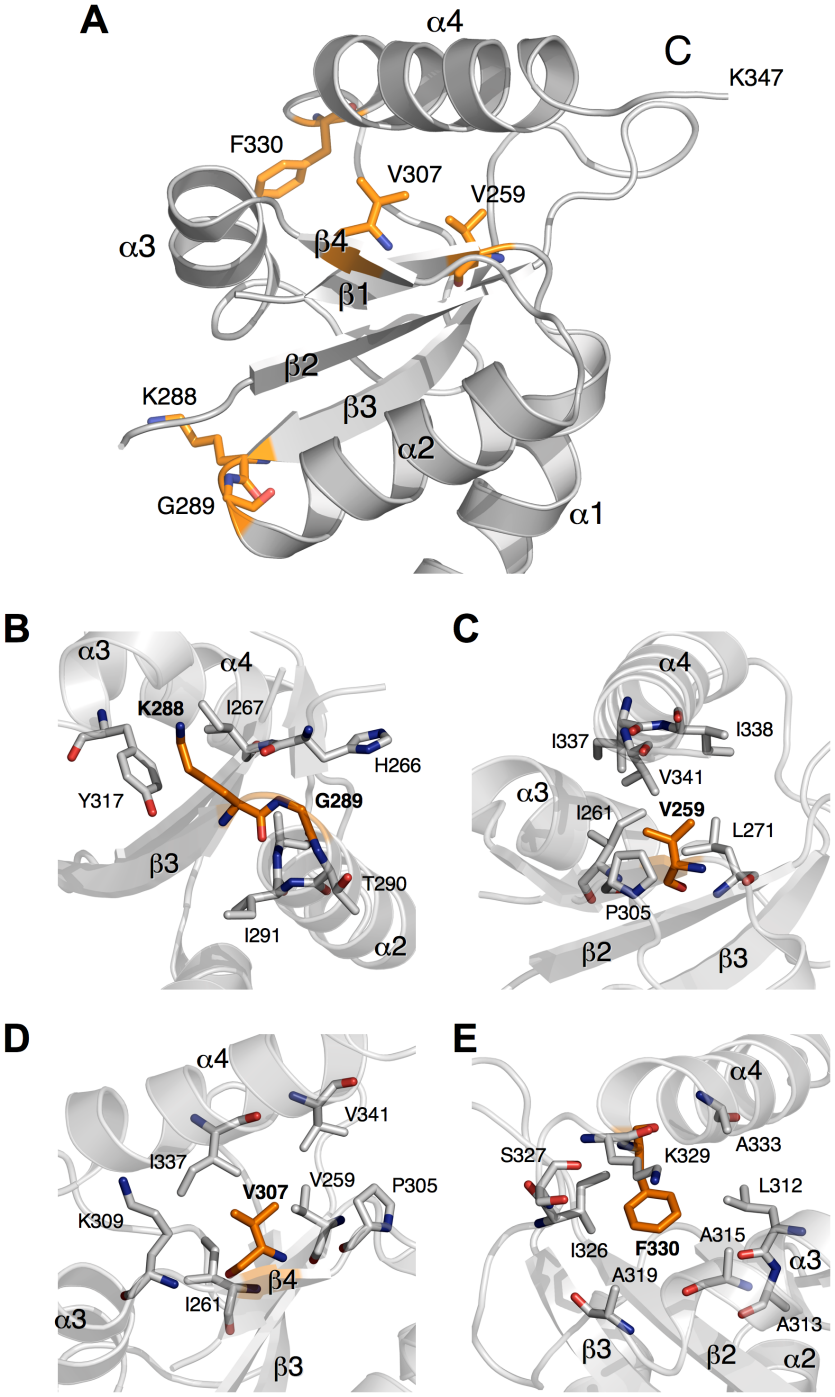
Mapping of the suppressor mutations in *Aquifex* FlhB_C_ (PDB accession code: 3B1S). (A) Ribbon diagram of *Aquifex* FlhB_C_ with the suppressor mutations shown as sticks. Panels (B) to (E) show close-up views of the mutated residues and their surroundings.

The positions of the suppressor mutations were mapped onto the structure of AquFlhB_C_ (Figure 6). Two frame shift mutations, V341 and F343, are positioned very close to the C-terminus of the molecule. It is difficult to predict how they could affect the whole FlhB_C_ structure. Residues V259 and V307 are located close to each other in a hydrophobic core between helix α4 and the β-sheet. Mutations in these positions, especially V259E with a change from a hydrophobic to a charged side-chain, are likely to destabilize the hydrophobic core and disrupt packing of the helix against the β-sheet. Residues K288 and G289 are located between strand β3 and helix α2. Deletion of these residues would disrupt helix α2 and interfere with the hydrophobic core formed by the β-sheet and helices α1 and α2. Mutation F330L may affect the hydrophobic surface between helices α3 and α4. Thus, most of the suppressor mutations (except the frame shift mutations) are located in or close to a hydrophobic core of the protein, and are likely to result in destabilization of its structure.

### Circular Dichroism and Stability Studies

Structural analysis suggests that the suppressor mutations could affect the stability of FlhB. To test this idea, *Aquifex* FlhB_C_ mutant proteins were purified and their stability was checked. First, we measured thermal stability of the proteins using a fluorescence-based thermal shift assay [18]–[20]. The melting temperatures were 63.7°C±0.5 for *Salmonella* FlhB_C_; 78.9°C±0.2 for *Aquifex* FlhB_C_ V259E; and 54.5°C±0.3 for *Aquifex* FlhB_C_ ΔKG (288–289). Thermal denaturation was not observed for the native *Aquifex* FlhB_C_ and for the mutants V307G, F330L, and frameshift V341, most likely because of the high thermal stability of these proteins (higher than 100°C - the temperature limit of the thermal shift assay).

**Figure 7:**
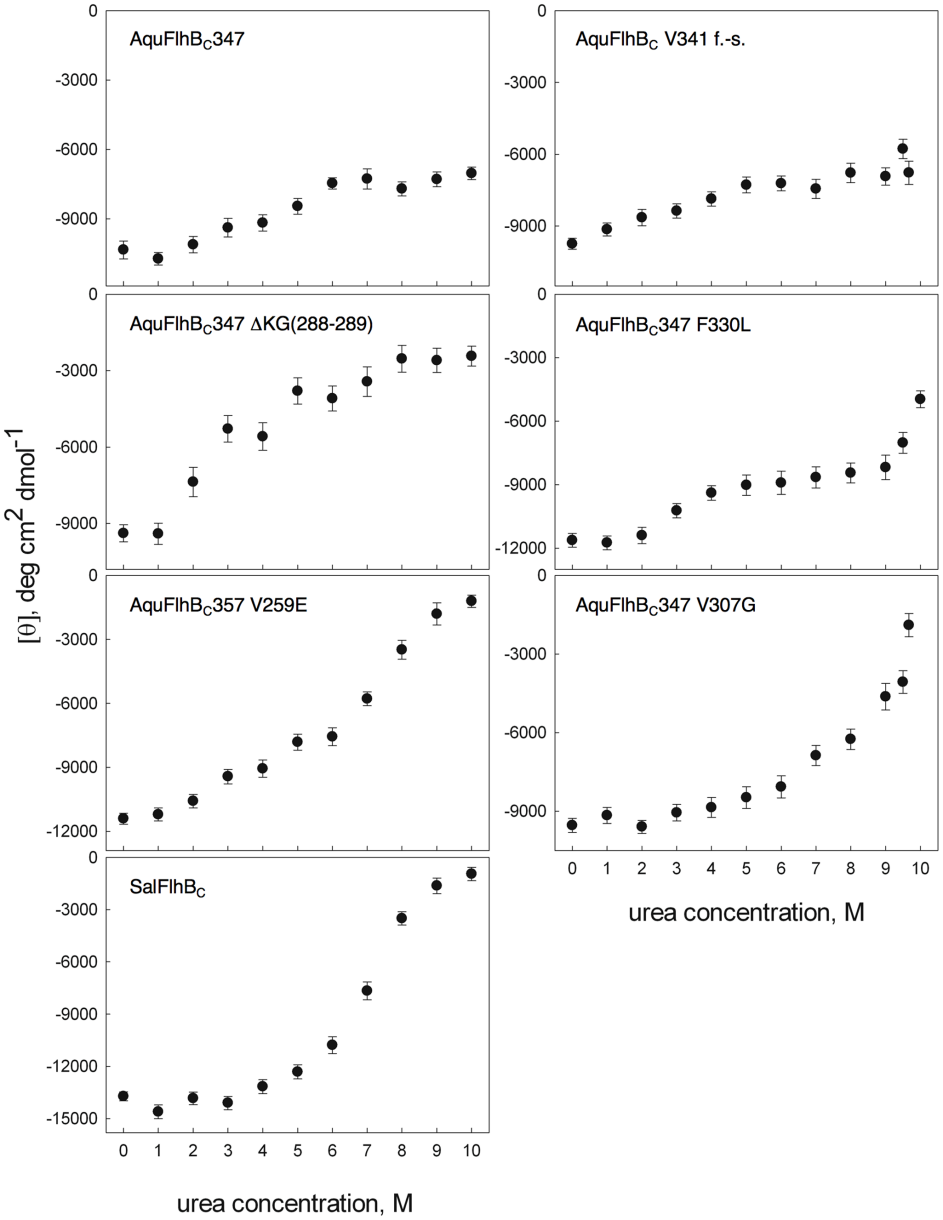
Urea denaturation of *Salmonella* and *Aquifex* FlhB_C_ and various *Aquifex* FlhB_C_ mutants. Protein unfolding was monitored by ellipticity at 222 nm.

The secondary structures of wild-type FlhB_C_ and mutants were calculated from their circular dichroism (CD) spectra. To perform analysis of the stability of the mutated AquFlhB_C_, we used chemical denaturation in urea. Monitoring of unfolding was done by circular dichroism spectroscopy. The dependence of mean residue ellipticity on urea concentration for wild-type and mutant *Aquifex* FlhB_C_ is shown in Figure 7.

The shapes of the titration curves of *Aquifex* FlhB_C_, wild-type and mutants, suggest that there are two transition states. There might be at least two cooperatively unfolding domains in AquFlhB_C_ with the midpoint of the first transition corresponds to 4 M urea, whereas the second domain stays folded in wild-type protein even at 10 M urea. These domains might correspond to the two fragments generated by the auto-cleavage of FlhB at the conserved NPTH sequence.

**Figure 8:**
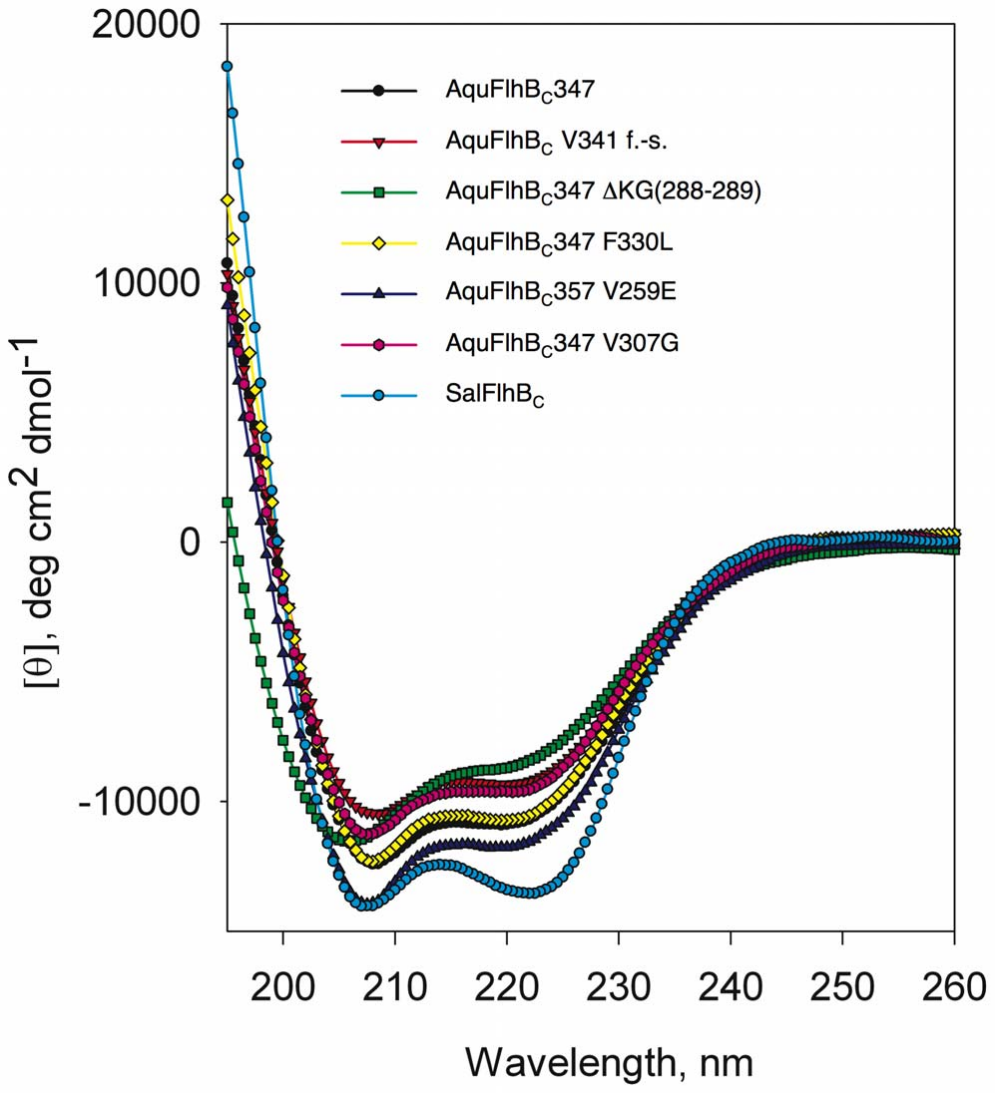
Circular dichroism measurements of *Aquifex* FlhB_C_, *Aquifex* FlhB_C_ mutants, and *Salmonella* FlhB_C_.

Of all the suppressor mutations, only the frame shift V341 had no effect on the secondary structure and stability: there was no difference in the CD spectra (Figure 8), and the unfolding curves (Figure 7) and the calculated secondary structure content (Table 1) between *Aquifex* FlhB_C_ and this mutant are similar.

**Table 1:** Secondary structure content of various FlhB_C_. Secondary structure content was calculated from the CD spectra using a multilinear regression [36].

Protein	α-helix, %	β-structure, %	β-turn, %	random coil, %
AquFlhB_C_347	28.5±0.1	28.7±0.1	5.2±2.9	37±1.8
AquFlhB_C_ V341 f.-s.	31.3±1.5	25.3±0.7	6.1±0.5	37.4±2.8
AquFlhB_C_347 ΔKG(288–289)	17.3±1.3	30.4±0.3	6.1±0.01	46.4±1.1
AquFlhB_C_347 F330L	32±3.1	26.1±1.3	4.45±1.1	37.5±0.01
AquFlhB_C_357 V259E	27.3±1.3	25.8±1.6	6.5±0.7	40.4±0.4
AquFlhB_C_347 V307G	28.4±1.8	27.7±1.0	4.6±0.3	39.4±0.4
SalFlhB_C_	40.8±0.7	14.4±0.6	9.9±1.5	34.9±0.2

Mutations V307G and F330L had no measured effect on the secondary structure. Mutation V259E caused a slight decrease of β-structure content and a corresponding increase of random coil content (Table 1). However, the effect of these three mutations on the stability of AquFlhB_C_ differed drastically. F330L and V259E decreased stability of the more stable second domain of AquFlhB_C_, V259E having the greater effect. For the V259E mutation, the transition midpoint is 8 M urea, and for F330L, 10 M urea. The V307G mutation increased stability of the first domain but decreased stability of the second domain to intermediate between the stabilities of F330L and V259E.

The ΔKG (288–289) deletion had the biggest effect on structure and stability: an almost 2-fold decrease in helical content with a corresponding increase in random coil content (Table 1). Stability of both putative domains substantially decreased, and with these mutations AquFlhB_C_ became completely unfolded at 8 M urea, the midpoint of transition is ∼3.5 M urea (Figure 7). This effect may be explained by the disruption of helix α2 as well as by the destabilization of an adjacent part of helix α1 when these two residues are deleted. This supports the conclusions based on the crystal structure analysis.

The range of effects seen for the suppressor mutations is consistent with the structural analysis. V307G and F330L, being on the protein surface, would be predicted to have less effect on stability. In contrast, the introduction of a charged Glu residue instead of Val (V259E) inside the hydrophobic core, or deletion of part of the structurally important helix α2 (ΔKG(288–289)) would be expected to have larger effects on stability.

### Extragenic Suppressor Mutation in *fliS*

**Figure 9:**
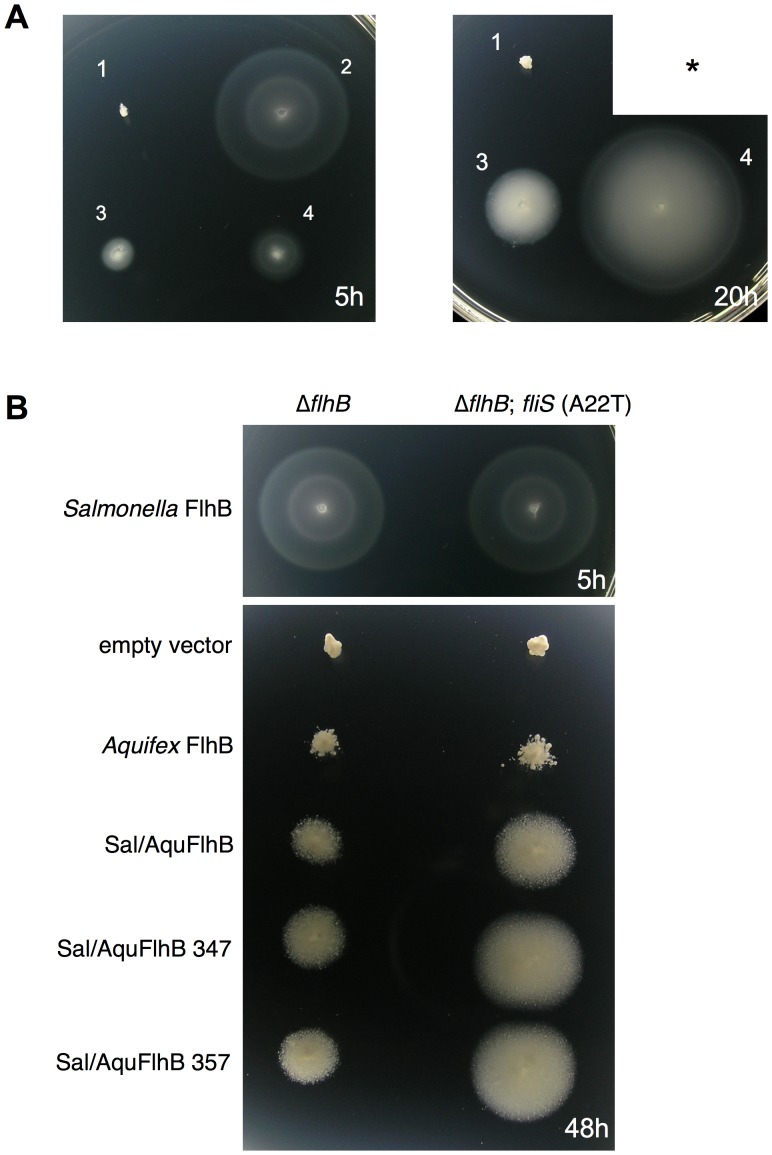
Effect of *fliS* mutation on motility of *Salmonella* cells. Cells were transformed with pTrc99A-based plasmids encoding various FlhB proteins. Soft agar plates were incubated at 30°C for the indicated time. (A) Motility of Δ*flhB Salmonella* strains producing Sal/AquFlhB F343 frame shift. One of the strains contains additional mutation in *fliS*. 1 - empty vector, 2 - wild type *Salmonella* FlhB, 3 - Sal/AquFlhB F343 frame shift, 4 - Sal/AquFlhB F343 frame shift plus FliS (A22T). The asterisk “*” marks the previous emplacement for the wild type strain that was removed because it would have overgrown after 20 hours. (B) Comparison of the motility of Δ*flhB* and Δ*flhB fliS*(A22T) *Salmonella* strains producing different FlhB variants.

We found that two suppressor mutants carried an identical mutation of the fusion *flhB* gene, frame shift F343. However motility of these strains was substantially different (Figure 9A). Therefore we concluded that one of the strains, CB351, must bear an additional extragenic mutation. Genomic DNA of this strain was isolated and sequenced. A single point mutation, which was responsible for the suppression effect, localized to the *fliS* gene and encoded a FliS A22T mutant. To further investigate the effect of this mutation on motility, *Salmonella* strain Δ*flhB fliS*(A22T) was constructed from strain MKM50. We found that the A22T mutation of FliS enhanced motility of *Salmonella* cells expressing *Aquifex* FlhB or different FlhB chimera (Figure 9B), although its suppression ability was much weaker than that of the intragenic FlhB suppressor mutations identified earlier. The FliS A22T mutant did not affect the motility with wild type *Salmonella* FlhB.

**Figure 10:**
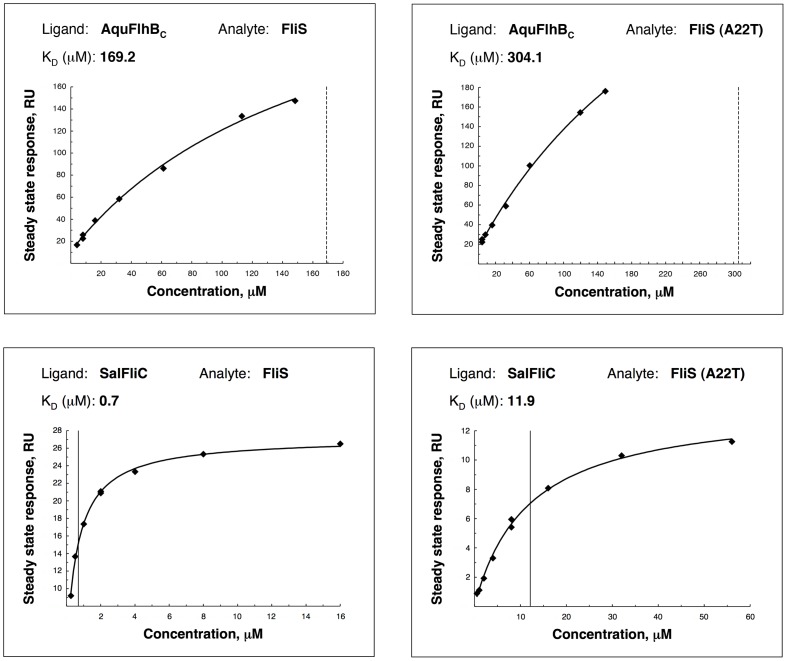
Steady-state surface plasmon resonance analysis of FliS binding to *Aquifex* FlhB_C_ and FliC. FliS (or FliS A22T) of various concentrations was flowed over the sensor surface with immobilized ligand (AquFlhB_C_ or FliC) in Biacore buffer (10 mM HEPES pH 7.4, 150 mM NaCl, 0.05% (v/v) Surfactant P20). Response at steady state was plotted against analyte concentration. K_D_ is measured as the protein concentration that gives response equal to 50% saturation.

The mutation of FliS that enhances motility of FlhB mutant cells suggests interaction between these two proteins. One of the possible effects of this mutation could be a change in the affinity of FliS to FlhB_C_. On the other hand, Ala22 lies in the region that directly contacts with FliC [21], the natural binding partner of FliS in *Salmonella* cells [22]. Obviously, mutation of this amino acid could affect FliS-FliC interaction. To check both possibilities, interactions between *Salmonella* FliS and *Salmonella* FliC or *Aquifex* FlhB_C_ were analyzed using surface plasmon resonance technique (Biacore, GE Healthcare). AquFlhB_C_, or *Salmonella* FliC, was amine coupled to a sensor chip and allowed to bind to flowing FliS and FliS A22T. A dissociation constant K_D_ was calculated using steady state analysis with 1∶1 binding model (Figure 10). There were some differences between affinities of AquFlhB_C_ to FliS and FliS A22T (K_D_ is 169.2 µM for wild-type FliS, and 304.1 µM for FliS A22T), however we cannot make any conclusions since the affinity for both cases was very low and as a result the estimation was not very confident. The affinity of FliS mutant A22T to FliC was greatly reduced in comparison with wild type protein (0.7 µM for wild-type FliS and 11.9 µM for FliS A22T). Thus mutation of FliS affects interaction between FliS and FliC, but not between FliS and FlhB.

## Discussion

FlhB is a key protein in the regulation of secretion by the type III secretion system. Homologues of FlhB were found in all type III secretion systems including *Aquifex*, one of the deepest-branching lineages within the bacteria having flagella. In this work we applied a new genetic approach, which allowed us to reveal functionally important properties of FlhB. Complementation properties of FlhB from two very distantly related organisms: *S. typhimurium* and *A. aeolicus*, have been investigated. We showed that Sal/AquFlhB chimeras (or the whole *Aquifex* FlhB) could not replace native FlhB in *Salmonella* cells. But functionality of the substituted protein could be restored to some degree by mutations that destabilize the protein. It is well known that proteins from hyperthermophilic organisms (like *Aquifex*) possess rigid structure at moderate temperature. For this reason thermophilic enzymes are not functional at normal temperature since they do not have enough flexibility to perform their function [23]–[25]. FlhB has been suggested to exist in two conformational states corresponding to two substrate specificity modes: rod/hook mode and filament mode [9], . The flexibility might be essential for transition from one state to another. The necessary degree of conformational freedom, in particular, might result from the autocleavage within the highly conserved NPTH sequence. In our experiments we could not detect any improvement in autocleavage for the suppressor mutations that improve the functionality of FlhB. However, while we cannot completely exclude possible effect of these mutations on FlhB autocleavage, we think that there could be other factors important for the protein function. One of such factors could be flexibility of FlhB molecule. It is also possible that the suppressor mutations in AquFlhB_C_ affect the binding of FlhB to other proteins of the type III secretion system. Investigation of this possibility is a subject for future research.

The conformation changes in wild-type *Salmonella* FlhB are catalyzed through interaction between FlhB_C_ and FliK. However, a *fliK* gene was not found in the *Aquifex* genome [26]. It could be that at extremely high temperatures flexibility of FlhB_C_ is enough to switch even without help of FliK. It is not clear whether *Aquifex* FlhB can recognize FliK the same way as *Salmonella* FlhB. Such recognition could be important for the function of *Aquifex* FlhB in *Salmonella* cells.

Proteins of the FlhB family exhibit significant variation in length (mainly because of differences at the C-terminus). *Salmonella* FlhB is longer than *Aquifex* by 33 residues. To check importance of these residues for function of the protein, we created chimera FlhB having C-termini identical to wild-type *Salmonella* FlhB. We found that addition of extra residues to C-terminus of chimera FlhB almost has no effect on motility of *Salmonella* cells. Moreover, one of the frame shift suppressor mutation (F343 frame shift) occurred in Sal/AquFlhB357. This chimera has a C-terminus native to *Salmonella* FlhB, however the C-terminus is apparently not functional and must be modified to restore protein activity. Surprisingly, the C-terminus created by the frame-shift mutation was identical to that found in several frame-shift mutants of *Salmonella* FlhB that suppress the phenotype of the *fliK* deletion [11], [13]. Currently existing ideas suggest that Δ*fliK* suppressor mutations of FlhB could change conformational flexibility of FlhB or its affinity to other proteins [8], [11], [27]. However, there is no clear understanding how frame shift mutations of FlhB could affect protein function. Apparently, the C-terminal part of *Salmonella* FlhB is involved in the secretion regulation specific for *Salmonella*. We strongly believe that there is something common between Δ*fliK* suppressor mutations of *Salmonella* FlhB mutations and the mutations we describe in this paper. Definitely, investigation of this relationship deserves more detailed study in the future.

One extragenic suppressor mutation we identified was found in the *fliS* gene. FliS is a chaperone, which specifically binds to flagellin (FliC), the major component of flagellar filament, and prevents its polymerization [21]. Presumably FliC-FliS complex is delivered to the export apparatus, where it must dissociate, since only FliC is translocated through the cell membrane [28], [29]. However details of FliS-FliC dissociation are not known. We found that the mutation of FliS (A22T) improves the motility of *Salmonella* cells expressing chimeric *flhB* genes while it decreases the affinity of FliS to FliC. These data suggests that FlhB might be involved in the process of FliS-FliC dissociation. Although we could not detect stable complexes between *Salmonella* FlhB and FliS-FliC or isolated FliS and FliC proteins *in vitro* (data are not shown), such interactions can be possible *in vivo* where FlhB works together with other proteins of type III secretion system. The cytoplasmic domain of FlhA, another membrane protein of type III secretion system, is known to interact strongly with FliS-FliC complex [30]. Whether FlhB also participates in this process releasing FliC prior to secretion is a question for future work.

## Materials and Methods

### Bacterial Strains, Plasmids and Growth Conditions

The bacterial strains and plasmids used in this study are listed in Tables 2 and 3, respectively. Bacteria were routinely cultured in Luria-Bertani Broth medium with continuous shaking at 37°C. For *Salmonella* strains, ampicillin was used in media at 100 µg ml^−1^, and for *E. coli* strains it was used at 50 µg ml^−1^. Tetracycline was used at 15 µg ml^−1^ where appropriate.

**Table 2:** Strains used in this study.

Strain	Genotype			Source/ref.
*Escherichia coli*				
NovaBlue	Recipient for cloning experiments			Novagen
BW25113	Plasmid pKD46 (Amp^R^)			CGSC7739^a^
BL21(DE3)	Host for overexpression from T7 promoter			Novagen
Rosetta™	Host for overexpression from T7 promoter			Novagen
*Salmonella enterica* serovar Typhimurium				
JR501	R^−^m^+^ for converting plasmids to *Salmonella* compatibility			FBS^b^ [39]
TT13206	LT7 *phoN51*::Tn*10*-*11*(Tet^R^)			SGSC3718^c^
SJW1103	Wild-type for motility and chemotaxis			FBS^b^ [40]
MKM50	Δ*flhB* null mutant			FBS^b^ [7]
CB351	Δ*flhB fliS22264*(*A22T*) carrying plasmid pTB342 (Amp^R^)			This study
VM001	Δ*flhB fliS22264*(*A22T*) derived from MKM50 (Δ*flhB*)			This study

a CGSC, *Escherichia coli* Genetic Stock Center, Yale University, USA.

b FBS, School of Frontier Biosciences, Osaka University, Japan.

c SGSC, *Salmonella* Genetic Stock Centre, University of Calgary, Canada.

**Table 3:** Plasmids used in this study.

Plasmid	Relevant characteristic	Ref.
pKD46	*λ*-Red genetic engineering plasmid; temperature-sensitive *ori* (30°C); Amp^R^	CGSC
pTrc99A-FF4	Modified pTrc99A expression vector; Amp^R^	[17]
pMM26	pTrc99A-FF4 derivative encoding wild-type *S. typhimurium* FlhB (1–383)	[9]
pTAB32	pTrc99A-FF4 derivative encoding wild-type *A. aeolicus* FlhB (1–350)	[32]
pTB334	pTrc99A-FF4 derivative encoding a FlhB chimera:	This study
	*S. typhimurium* FlhB_1–218_-*A. aeolicus* FlhB_213–350_	
pTB335	pTrc99A-FF4 derivative encoding a FlhB chimera:	This study
	*S. typhimurium* FlhB_1–218_-*A. aeolicus* FlhB_213–340_-*S. typhimurium* FlhB_347–383_	
pTB336	pTrc99A-FF4 derivative encoding a FlhB chimera:	This study
	*S. typhimurium* FlhB_1–218_-*A. aeolicus* FlhB_213–350_-*S. typhimurium* FlhB_357–383_	
pTB337	pTrc99A-FF4 derivative encoding a FlhB chimera:	This study
	*S. typhimurium* FlhB_1–218_-*A. aeolicus* FlhB_213–341_-VQKEKGLRLIGSSRVDLQACKLGCFGG	
pTB338	pTrc99A-FF4 derivative encoding a FlhB chimera:	This study
	*S. typhimurium* FlhB_1–218_-*A. aeolicus* FlhB_213–340/F330L_-*S. typhimurium* FlhB_347–383_	
pTB339	pTrc99A-FF4 derivative encoding a FlhB chimera:	This study
	*S. typhimurium* FlhB_1–218_-*A. aeolicus* FlhB_213–340/V307G_-*S. typhimurium* FlhB_347–383_	
pTB340	pTrc99A-FF4 derivative encoding a FlhB chimera:	This study
	*S. typhimurium* FlhB_1–218_-*A. aeolicus* FlhB_213–340/Δ(288–289)_-*S. typhimurium* FlhB_347–383_	
pTB341	pTrc99A-FF4 derivative encoding a FlhB chimera:	This study
	*S. typhimurium* FlhB_1–218_-*A. aeolicus* FlhB_213–350/V259E_-*S. typhimurium* FlhB_357–383_	
pTB342	pTrc99A-FF4 derivative encoding a FlhB chimera:	This study
	*S. typhimurium* FlhB_1–218_-*A. aeolicus* FlhB_213–343_-RFTPAGNVLHNLRTFRCQKRWIL	

CGSC, *Escherichia coli* Genetic Stock Center, Yale University, USA.

### Plasmid and Strain Constructions

The oligonucleotides used in construction of plasmids and strains are listed in Table 4. The *flhB* chimera gene encoded by plasmid pTB334 was derived from *S*. *typhimurium* and *A. aeolicus* genomic DNA by PCR-driven overlap extension, similar to previously described [31], [32]. For the swapping of domains within genes carried on plasmids, the plasmid was first made linear at the desired location using PCR with a thermophilic DNA polymerase lacking strand-displacing activity. The DNA fragment to be inserted was also derived by PCR, and contained ends homologous to the linear plasmid. The two DNA fragments were then fused to recreate circular plasmid DNA, using an In-fusion cloning kit (Clontech Laboratories, Inc., USA).

**Table 4:** Oligonucleotides used in the strain and plasmid constructions.

Primer name	Sequence (5′ to 3′)^a^
5′-*Sal*-*flhB*	CATATGGCAGAAGAGAGCGACGAC
3′-*Sal*-*flhB*-1-218	GACATCATTATCTTTTTCAGGTGGCTAAAGATCTGG
5′-*Aqu*-*flhB*-213-350	CTTTAGCCACCTGAAAAAGATAATGATGTCGAGAAGGGAATTG
3′-*Aqu*-*flhB*	CTAGAGGATCCTATTAGGCGTAAACC
5′-pMM26-Linear-347	GTCTGGCAGCTTAAACGCTGG
3′-pMM26-Linear-218	TTTCAGGTGGCTAAAGATCTGG
5′-*Aqu*-*flhB*-213	TTTAGCCACCTGAAAAAGATAATGATGTCGAGAAGGG
3′-*Aqu*-*flhB*-340	TTTAAGCTGCCAGACGTAGGCTATTATTTCCGCTACGG
5′-pMM26-Linear-357	GGCGGGCAACGTCCTCCAC
3′-*Aqu*-*flhB*-350	AGGACGTTGCCCGCCGGCGTAAACCTTTTTCTTTTTGAAC

a Restriction sites are underlined.

To genetically engineer strains, bacteriophage *λ*-Red-based recombination was used [33]. Genomic DNA was first prepared from *E. coli* strain TT13206, which contains a Tn*10-11* element on the chromosome, conferring tetracycline-resistance [34]. Tetracycline-resistance (*tetRA*) cassettes, flanked by ends homologous to the intended chromosomal target site were obtained by PCR using TT13206 DNA as template. These cassettes were then inserted into the chromosome of the desired strain by homologous recombination. Following this, it was possible to counter-select against tetracycline-resistance on medium containing fusaric acid, and replace the *tetRA*-cassette with a PCR product containing ends homologous to the target site.

### Soft Tryptone Agar Motility Assays and Isolation of Motile Suppressor Mutants

Soft tryptone agar contained 0.35% (w/v) agar, and was used in motility assays at 30°C [35]. Freshly transformed cells were inoculated as colonies directly into soft tryptone agar. To test for dominant effects, 0.1 mM IPTG was included in the agar plates. To isolate motile suppressor mutants, 10 µl of an overnight culture were inoculated as a streak into soft tryptone agar. After incubation, suppressor mutants were purified from the edges of outgrowths generated by motile cells.

### Protein Purification

Purification of *Aquifex* FlhB_C_, *Salmonella* FlhB_C_ and the mutated AquFlhB_C_ proteins was done as described previously [14]. Purification of the F343 frame shift mutant was not successful; therefore this mutant was not used for further experiments.

### Thermal Shift Assay

TSA experiments were carried out in triplicates using a 7500 Real-Time PCR System (Applied Biosystems, Inc.). The reaction mixture contained 0.2 mg ml^−1^ purified protein, 5× SYPRO Orange dye (Molecular Probes, Invitrogen) in 10 mM K-phosphate pH 6.1, 50 mM NaCl. Reactions were heated from 20 to 95°C with a rate of 1°C per minute. Protein unfolding was monitored by detection of changes in fluorescence of SYPRO Orange dye. Fluorescence intensities were plotted as a function of temperature, and the midpoint of the unfolding transition was taken as an estimation of the melting temperature.

### Circular Dichroism Spectroscopy and Chemical Denaturation

Far-UV CD spectra were measured using an Aviv model 400 spectropolarimeter (Lakewood, NJ) in 1 mm cuvettes in 10 mM K-phosphate pH 6.1, 50 mM NaCl. Changes in helical content during urea denaturation were monitored at 222 nm. Secondary structure content was calculated from the CD spectra using a multilinear regression [36]. The errors represent standard deviations for the secondary structure content calculated from the CD spectra of different preparations of the same proteins. The changes that we describe as significant ones based on the calculations of the secondary structure are confirmed by the shifts of the spectra intersection with the X-axis to lower wavelengths, which is characteristic for the increase of random coil content.

### Biacore Analysis

All analyses were carried out on a Biacore T100 (T200 Sensitivity Enhanced) (GE Healthcare). Ligand AquFlhB_C_ or FliC was immobilized on a CM5 chip by amine cross-linking. Analyte FliS or FliS (A22T) of various concentrations in binding buffer (10 mM HEPES pH 7.4, 150 mM NaCl, 0.05% (v/v) Surfactant P20) was passed over the sensor surface at a flow rate of 30 µl min^−1^ for 12 min. Dissociation of the sample was monitored in binding buffer for 12 min. Surface was regenerated using 10 mM Glycine pH 2.0. All measurements were performed at 25°C. Data were analyzed by Biacore T200 Evaluation software using a 1∶1 binding model.

### Sequence Alignment

Protein sequences were aligned using ClustalW2 [37], [38].
